# Authors’ Reply: Predicting the Emergency Department Patient Journey Using a Machine Learning Approach

**DOI:** 10.2196/73342

**Published:** 2025-12-19

**Authors:** Dhavalkumar Patel, Eyal Klang, Prem Timsina

**Affiliations:** 1 Icahn School of Medicine at Mount Sinai, Institute for Healthcare Delivery Science, 7400 River Rd #231, New York City, NJ, 10017, United States, 1 2018794854

**Keywords:** Bio-Clinical-BERT, term frequency–inverse document frequency, TF-IDF, hospital resource management, resource management, logistic regression, retrospective analysis, large language model, emergency department, health informatics, patient care, language model, machine learning, hospitalization, deep learning

We appreciate the letter by Kovoor et al [1] referencing our recent article on predicting hospitalizations from nurse triage notes [2]. We congratulate the authors on their work using XGBoost (extreme gradient boosting), random forest, and logistic regression to predict multiple emergency department (ED) outcomes, including prolonged length of stay and inpatient admissions. Their integration of systemic factors, such as bed occupancy, underscores how operational data can enhance model performance.

One key area of interest is the integration of structured electronic health record data, such as vitals or laboratory values, with free-text triage notes. Our experience suggests that combining narrative descriptions with numerical features can yield deeper insights than either source alone, capturing both clinical context and objective measurements. Furthermore, recent studies point toward improved performance when text-based features are complemented by relevant structured data, offering a richer perspective on patient acuity and ED workflow [3].

Another promising direction involves large language models (LLMs). We have explored the use of GPT-4 for predicting ED admissions using real-world triage scenarios. Our approach used two methods: a naive application of the LLM and an augmented approach incorporating retrieval-augemented generation examples and probabilities derived from established machine learning models. Although a naive LLM approach might be outperformed traditional approaches, providing relevant clinical examples and numeric predictions can significantly enhance its performance, narrowing the gap. This synergy between LLMs and conventional machine learning could pave the way for more adaptive and interpretable decision support tools in the ED [3].

We agree with the authors that incorporating systemic variables can enrich predictive power and look forward to further exploration in real-time settings. Our findings suggest that simpler methods can be effective in resource-limited environments. The authors’ demonstration that XGBoost excels in their cohort supports the evolving role of various algorithms across diverse health systems.

We appreciate their contribution and share their view that ongoing validation and broader integration of machine learning can aid ED decision-making.

## Abbreviations

ED: emergency department
LLM: large language model
XGBoost: extreme gradient boosting
